# Sanitation behavior among schoolchildren in a multi-ethnic area of Northern rural Vietnam

**DOI:** 10.1186/1471-2458-12-140

**Published:** 2012-02-21

**Authors:** Le thi Thanh Xuan, Luu Ngoc Hoat, Thilde Rheinländer, Anders Dalsgaard, Flemming Konradsen

**Affiliations:** 1 Department of Environmental Health, Institute for Preventive Medicine and Public Health, Hanoi Medical University, Hanoi, Vietnam; 2 Department of Biostatistics and Medical Informatics, Institute for Preventive Medicine and Public Health, Hanoi Medical University, Hanoi, Vietnam; 3 Department of Veterinary Disease Biology, University of Copenhagen, Copenhagen, Denmark; 4 Department of International health, Immunology and Microbiology, University of Copenhagen, Copenhagen, Denmark

**Keywords:** Sanitation use, Behavior change, Schoolchildren, Vietnam, Multi-ethnic

## Abstract

**Background:**

In Vietnam, efforts are underway to improve latrine use in rural and remote areas with particular focus on increasing coverage of sanitation in schools. However, there is a lack of information on how the school program affects latrine use by schoolchildren and at community level. This paper analyzes sanitation use among schoolchildren in a multi-ethnic area to inform future school-based sanitation promotion programmes.

**Methods:**

A combination of quantitative and qualitative methods was applied during a 5 months period in six primary and secondary schools and in the homes of schoolchildren in four different ethnic villages in Northern rural Vietnam. Using a structured questionnaire, 319 children were interviewed face-to-face to collect quantitative data. Qualitative methods included extensive observations at schools and in the homes of 20 children, a single day's diary writings of 234 children, in-depth interviews with children (20), their parents (20) and school staff (10), and focus group discussions with parents (4) and teachers (6), and picture drawing with children (12).

**Results:**

All surveyed schools had student latrines. However, the observed schoolchildren most commonly urinated and defecated in the open. Main barriers for latrine use included inadequate number of latrines, limited accessibility to latrines, lack of constant water supply in latrines and lack of latrine maintenance by school management. Programs promoting latrine use for children were not conducted in either schools or communities and were not established as a preferred social norm in such settings. Children perceived existing school latrines as unappealing and expressed a wish to have basic, functional, clean, and colorful school latrines with privacy.

**Conclusions:**

The paper shows that the current school based sanitation promotion is insufficient to change sanitation behavior of school children irrespective of their ethnicity. It is important that schools, households and communities work more closely together to increase use and uptake of latrine use among schoolchildren. Also, the contractors of latrine facilities must work more closely with local school management when constructing latrines, including identifying location, design and appropriate systems of water supply. A separate budget needs to be allocated to allow the school to maintain the sanitation infrastructure and keep it hygienic and appealing for users.

## Background

It is becoming increasingly difficult to ignore the serious consequences that open defecation has for school-aged children, e.g. high risk of hygiene-related morbidity and mortality [1-3]. School-based sanitation promotion (SBSP) is considered to be feasible and can bring both health and non-health related benefits to children, such as maintaining high school attendance, promoting gender equity and establishing life-long healthy practices [4-6]. In addition, children could be "agents of change" impacting the behavior and practices of their family and community at large [2].

Previous studies have identified factors hindering latrine use by school children. Factors such as poor maintenance, smelly and dirty latrines [7,8], lack of sanitation facilities [5], overcrowding [9], and financial management [10] play a role on whether children will use the latrines. School staff was unable to teach children basic hygiene if the school did not have a sufficient number of latrines, lacked toilet paper and was not kept adequately clean [5,11-13]. Younger children, especially, felt uncomfortable using latrines in such conditions [14,15].

School-based sanitation promotion has been a major public health priority in Vietnam since 1986[16]. In 1998, a national strategy of Rural Water Supply and Sanitation (RWSS) was formulated. In 2000 a government National Target Program (NTP) for RWSS was approved, with the aim of providing school latrines for all rural schools by 2010. According to the NTP, 42% of Vietnam's 35,500 schools had latrines by the end of 2005 [17]. Standards on school health, including number of school latrines, have been issued by the Ministry of Health [18] and the Ministry of Education and Training [19]. In 2004, a standard for child-friendly latrines was developed with UNICEF support [20,21]. In 2008, decision 1486/QD-BGDDT was issued by the Ministry of Education and Training for the standard design of school latrines in kindergarten, primary and secondary schools [22]. Since 2010, school latrines are under the management of the Education Department [23].

In Vietnam, children normally begin primary school at the age of six and secondary school at the age of 11. In 2002, it was decided that hygiene education should no longer be a separate module in the curricula at primary schools, mainly because of the need to concentrate more on academic subjects [4]. Currently, education on the health and environmental consequences of open defecation and introduction to different types of latrines are only highlighted in a one-hour session in the Grade 3 curriculum under the subject "Nature and Society" [24].

Despite the development program and the recommendation to install standard latrines, the quality of school sanitation remains poor, in particular in the mountainous areas where schools often serve marginalized ethnic minority groups (EMG) [25-28]. This was documented in 2006 by a nationwide baseline survey on environmental sanitation and hygiene including 966 schools in rural Vietnam. The survey showed that 72.7% of the schools had latrines while at 21.3% of schools (with or without latrines), the children defecated in forests, gardens, fields, beaches and along streams and rivers. In addition, it was noted that teachers and school cleaners did not closely supervise student defecation practices [25]. It also revealed that we still lack detailed knowledge about sanitation behavior among school children in resource poor communities of Vietnam and their perceptions on using latrines [25,29]. There is also a common perception in Vietnam that EMGs have poor sanitary practices when compared with the majority population, and, therefore, an expectation that schoolchildren in remote areas where EMGs predominate would behave differently when using school latrines.

This study was conducted in school and home settings to analyze sanitation behavior and perceptions about using latrines among schoolchildren in a multi-ethnic area of two rural communes of Northern Vietnam with the aim of informing future school-based sanitation promotion programs.

## Methods

### Field sites

Two rural communes in a Northern Province of Vietnam were selected as study sites because they presented pilot sites for a range of sanitation and hygiene initiatives in the 2^nd ^phase (2006-2010) of the NTP-RWSS, including school-based sanitation activities. Both communes shared a mixed topography of mountainous highland areas with scattered villages and more densely populated lowland valley areas. The population of approximately 10,000 lives in 39 villages. Eighty percent of the population belongs to ethnic minority groups (EMGs), including the Dáy, Tày, Dao, Xa phó, H'Mong and Hoa groups, of which the Xa phó, Dao and H'Mong traditionally live in the highlands. Children in the communes attend primary school from the age of six and secondary school from the age of 11. The primary school system in the two communes has a main school located in the communal center and smaller branch schools located in villages. All schools in the study commune are government-owned and staffed by teachers from the majority ethnic group of Vietnam (the Kinh). Most children in lowland areas of the communes live in single-generation households, while many children from the highland live in extended families of two or three generations. All families live off agricultural activities. Many parents spend long days in the fields, especially in the highland areas where fields are located far away from home and agriculture is very labor intensive.

### Methods and materials

As hygiene behavior is a complex area to study [30], a mixed approach combining quantitative and qualitative methods was followed, with triangulation across different means of data collection and sources of information to improve the validity of the findings [31,32].

This research draws on both school and household-based data (Table 1). Six schools in two studied communes were selected to represent a diversity of school types (four primary and two secondary schools) and school areas (five schools located in the lowland and one smaller branch in the highland). Four villages from the two studied communes, one each of the four ethnic groups of Kinh, Tày and Dáy (all lowland) and Xa phó (highland) were selected for a household-based study in the homes of 24 schoolchildren.

**Table 1:** Methods applied in the study

**School no**.	School type	School area	Ethnic group of student	No. respondents for school based QRE (n = 319)	School observation	School study (interview)	Number of child households for home study
1	Primary	Low land	Day	32	1 week	1 Principal, 1 FGD with 5 teachers	4

2	Primary	Low land	Tay, Kinh	92	1 week	1 Principal, 1 FGD with 5 teachers, 1 FGD with 4 parents	4

3	Primary	Low land	Tay	32	1 week	1 Principal, 1 FGD with 5 teachers	4

4	Primary	Highland	Xa Pho	20	1 week	1 Principal, 1 FGD with 5 teachers, 1 FGD with 4 parents	4

5	Secondary	Low land	Kinh, Tay, Day, Xa pho, Dao	66	1 week	1 Principal, 1 FGD with 4 teachers, 1 FGD with 8 parents	2

6	Secondary	Low land	Kinh, Tay, Day, Xa pho	77	1 week	1 Principal, 1 FGD with 4 teachers, 1 FGD with 4 parents	2

#### School-based study

The field component of the school-based study was carried out over a six week period, from September to mid-October 2008; 319 children responded to a structured interviewer-administered questionnaire survey during school time, with a 100% response rate (Table 2). Questions explored children's latrine use pattern and differences considering age, gender and ethnic group. The selected children were from grades 1, 4 and 7 and were assumed to represent different levels of exposure to school-based sanitation promotion activities. The questionnaire was piloted among a group of 10 children and then revised before use. The questionnaire was answered by individual children in a separate and private location within the school premises. The questions concerning sanitation use included: if their household owned a latrine, where they had urinated the day before, and where they had defecated the day before. Multiple answers to the questions concerning defecation and urination were possible. All questions in the questionnaire referred to the previous school day. In addition, 234 school children from grades 4 and 7 who had answered the questionnaire were later asked to record their detailed activities, including sanitary behavior of the previous school day in a diary. To explore the children's perception of how a nice school latrine would look, 12 children from grades 4 and 7 were asked to draw a picture of their "dream school" including "a dream school latrine" [33].

**Table 2:** Main characteristic of 319 schoolchildren in the study

Variable	Frequency	Percent (%)
**School type**		

Secondary school	143	44.8

Primary school	124	38.9

Satellites	52	16.3

**Grade**		

Grade 1	62	19.4

Grade 4	114	35.7

Grade 7	143	44.8

**Sex**		

Male	165	51.7

Female	154	48.3

**Ethnicity**		

Kinh	7	2.2

Tay	163	51.1

Xa Pho	33	10.3

Day	116	36.4

Number of family members	319	5.5 ± 1.5

Household own latrine	174	54.0

**Household assets**		

Having TV	246	77.0

Having radio	94	29.0

Having bicycle	177	55.0

Having motor-bicycle	169	53.0

In addition, in-depth individual interviews focusing on hygiene and sanitation activities as well as the maintenance and management of school latrines were conducted with 6 school principals and 4 school gatekeepers. Ten focus group discussions (FGDs) were conducted, including four with 20 school parents and six with 28 head teachers. FGDs with teachers and parents followed a guide and focused on child-based latrine promoting programs in the community and school settings and on how teachers and parents supported the children in using latrines in the school context.

Furthermore, over a period of 1 week, intensive observations were carried out at each of the six schools included in the study. Systematic observations were conducted to collect information about the hygiene, infrastructure and maintenance of the school latrine. Open observations were conducted in class rooms and school yards to collect data on its school-based sanitation program activities and to observe children's use of latrines and other sites for urination and defecation when they were at school.

#### Household-level study

In order to learn more about child sanitation behavior in the home environment, 20 households (eight in highland and 12 in lowland) from four villages representing four different ethnic groups were selected and studied during July-August and October-November, 2008. The households included two Kinh, eight Xa phó, six Tày and four Dáy families.

The selected households had one or more children studying either in grade 1 (six children), grade 4 (nine children) or grade 7 (nine children) at one of the six selected schools, including 10 males and 14 females. The selected households also presented different latrine types.

Extensive observations were conducted in households during 76 observational days. Three to five days were spent in each of the 20 households starting from early morning around 6 AM to evening around 7-8 PM. Observations focused in particular on sanitation and hygiene practices of the child. In-depth interviews were conducted with all 24 children and 20 of their parents. The interviews with children focused on their perception of latrine use and how they were supported in using a latrine, while parent interviews focused on how children were supported in using a latrine at community level.

### Data processing, validation and analysis

All interviews were conducted in Kinh national language and carried out in a private area, either at school or at home, by a member of the research team composed of the first author and four research assistants. All interviewees were able to speak the national language (Kinh), except for 12 schoolchildren of grade 1 in a highland commune who were supported by a local translator. All interviewees were verbally informed about the study and their consent was obtained prior to the interview, including children. For each child, we obtained parental consent verbally before conducting any activities with the children. The study was approved by the Ethical Committee of the National Institute of Hygiene and Epidemiology, Ministry of Health, Vietnam and the relevant provincial, district, commune, schools and village authorities. Confidentiality and anonymity were guaranteed.

The questionnaire data was checked for accuracy in the field by the principal researcher before being entering into EPI-INFO 6.0 by the research team and descriptive analyzed using STATA statistical software (version 10.0). Frequency and percentages were cross-tabulated by gender, ethnic groups, age groups, school types (primary and secondary) and school location (highland and lowland) for the answers given by school children in relation to the three options for urination and defecation (open, school latrine and home latrine use) (Table 3).

**Table 3:** Latrine use pattern of 319 schoolchildren in two rural communes of Northern Vietnam

Variable	Total of respondent	Where did you urinate yesterday?*				Where did you defecate yesterday?*			
		Open urination	School latrine	Home latrine	Total of responses	Open defecation	School latrine	Home latrine	Total of responses
**Aggregated**	319	55%	18%	45%	378	53%	3%	44%	318
**Gender**									
Male	165	55%	22%	48%	207	53%	3%	44%	165
Female	154	54%	14%	43%	171	54%	2%	44%	153
**Ethnic groups**									
Kinh-Tày	170	44%	19%	54%	199	45%	3%	52%	171
Xa phó	33	52%	18%	48%	39	45%	0	45%	30
Day	116	72%	17%	32%	140	67%	3%	31%	117
**Grade**									
Grade 1	62	63%	5%	27%	59	60%	2%	31%	57
Grade 4	114	52%	12%	45%	124	54%	4%	42%	113
Grade 7	143	53%	29%	54%	195	50%	2%	51%	148
**School type**									
Secondary	143	53%	29%	54%	195	50%	2%	51%	148
Primary	176	56%	10%	39%	183	56%	3%	38%	170
School area**	52	46%	10%	46%	53	48%	2%	44%	49
lowland branch	32	44%	9%	41%	30	53%	3%	41%	31
highland branch	20	50%	10%	55%	23	40%	0	50%	18

* The% of respondents who had multiple responses

**We calculated for only branch to see the difference between lowland and highland

All in-depth interviews and FGDs were tape-recorded and the recordings transcribed into *ad verbatim *Vietnamese text by the research assistants. Data was analyzed by the principal researcher using the content analysis approach of qualitative data [34,35]. Analysis of data was performed in two steps: the first step was initiated in the field by the principal researcher when establishing tentative analytic concepts from observations, impressions, interviews and conversations. The second step comprised of a literature review and coding of all interview transcripts, including forming themes and expanding on analytical concepts. Themes included existing themes from the interview guides as well as emerging themes, including child-based sanitation promotion activities; physical environment of latrines, accessibility and hygienic conditions of both school and home latrines; child's perception of latrines; management of latrine at home and in schools and promotion of latrine use at schools and at home. Illustrative quotes were translated into English by the first author.

## Results

### Child-based sanitation promotion

From interviews with the principals and teachers at the six selected schools it was clear that the schools followed the national curriculum related to hygiene and no separate formal component on hygiene education and sanitation was included in the curricula.

However, 6 weeks of observations at the six schools did identify a number of general environmental hygiene promotion activities taking place outside the classroom across all schools. In six schools, the emphasis was on teaching children practical skills such as daily cleaning of classroom and weekly cleaning of schoolyards. In one secondary school, students participated in weekly cleaning of the school latrine and in keeping the road leading to the school free from garbage. During the observations and from interviews with schoolchildren it was clear that no practical instructions were given to the children on how to use a latrine.

Based on in-depth interviews with the parents and responses from the children, no child-focused sanitation promotion activities were identified as ongoing at the community level. Village health workers and village heads were identified as the main actors responsible for community-based sanitation promotion. However, none of them performed any such activities targeting children during the time of the study [36].

### Latrine infrastructure and availability at school

All six schools had latrine facilities which were located within the school compound and all had gender segregated compartments. Each compartment was designed with separate areas covered by a roof for defecation and separate urinals without a roof.

The location of the latrine depended on the size of the school compound. It was observed that two schools with large compounds had their latrines located in a secluded area where the earth path to the latrine was slippery, especially in the rainy season. In the remaining four schools with a small compound, the latrines were located nearby the classroom with a concrete path leading to the latrine. Only two out of the six schools had separate latrines for the staff. In general, each school had only one latrine with two or four pits serving between 67 and 247 children. At all schools, children were allowed to use the latrine during class times.

Observations also showed that the school latrines lacked essential materials and items allowing for proper use. For example, no anal cleansing materials, soap or waste baskets were seen at any of the schools.

Observations at the individual schools showed that the latrines were inadequately cleaned and therefore appeared dirty and in poor hygienic condition. The latrines had a strong smell of urine, especially at the end of the school day, the floor was dirty from soil and waste materials, and flies were abundant. Four of the six schools were found to have feces continually present on the latrine floor during the week of observation.

### Latrine infrastructure and availability at home

It was observed that children had limited opportunities to use a latrine at home, with only eight out of 20 households visited having a latrine. Four of these had the latrine connected to a septic tank with a structure made of a concrete roof, walls and floor. All latrines were located a few meters away from the main house. All households with a latrine connected to a septic tank had a door at the entry to the latrine and water, soap and toilet paper were available. The other four households also had a latrine but with very simple construction, including three pit latrines and one over-hanging fish-pond latrine. These latrines were made of local material and did not have a door. No water was available and the paths leading to these latrines was narrow, slippery and observed to be difficult to access and use in the rainy season, especially for small children. During the home visits, it was observed that the latrines floors were littered with fecal matter, soil, waste and a lot of flies present. Only two of the latrines were found to be well maintained.

### Contextual appropriateness of latrine

Through observation at school and home, we found that the residents, including children, preferred to urinate and defecate in the open. There seemed to be no stigma associated with this traditional practice and open urination and defecation were socially acceptable at village level. Regarding open defecation, the parents and children commonly stated that "everybody here does the same, all villagers practice similarly" (*ai cũng đi như thế, cả làng đi như thế*). Therefore, based on our observations, "hygienic" defecation means to either defecate into the stream that will carry the feces away, or dogs took care of children's feces. This attitude might be one reason for not having a latrine at home.

According to several parents in highland as well as lowland, *the concept of a latrine is perceived to come from the urban areas or the city where living conditions are better-off or considered as part of a 'civilized world' (thế giới văn minh)*. A septic tank is perceived as a sign of a better-off family in the village who has regular visitors and represents a higher social class in the local area.

Responding to the question of why many children practiced open urination and defecation in the village, a mother of grade 4 female student replied that "*Go to the stream.... Local people and children here do the same, go to the stream"*.

Through observation and interviews with local people, we found that it was collective perception that open defecation is common practice at the village level.

The interviewer: Well, which latrine the people here like to use?

The female parent: (laugh)

*The male parent 1: generally speaking, if you go to the rural, you like to*....

The male parent 2: open, like to go in open (tự do, đi tự do)

(FGD with parents of secondary student)

### Latrine use by schoolchildren

Table 3 describes the latrine use pattern of the schoolchildren. About half of the children reported urinating (55%) and defecating in the open (53%). Out of 319 schoolchildren responding to the question "Where did you defecate yesterday?", 170 children reported 'in the open', 140 'in the home latrine' and only eight (3%) used the school latrine. Observations made at the school settings and during child interviews confirmed the low use of the school toilets for defecation. At the six schools, latrine use practices were observed for more than thousand children and only 19 children were seen to use the school latrine for defecation. Furthermore, only seven of the 24 children selected for home visits and in-depth interviews reported ever having defecated in the school latrine. The analysis of the children's diaries describing their sanitation practices over a one day period also indicated that latrine use was not a common practice among the children. Only 23 out of 243 children reported having used a latrine either at home or at the school. The overwhelming preference for open defecation was clearly supported by our observations in the community and household settings.

In-depth interviews with children indicated that they found it more comfortable and convenient to defecate in the open, not least because of more fresh air. A grade 7 male child explained *"I prefer defecating in the bush because it is a cool and convenient place"*. Another grade 7 female child explained why she preferred to defecate in the stream *"I like to defecate into the stream because it will make it [excreta] flow away"*.

Results from the school-based questionnaire survey found limited differences in latrine use between boys and girls (Table 3). Likewise, no differences were found among children attending schools located in lowland as compared with highland areas. However, there was a difference in latrine use among children attending secondary and primary schools, with older children reporting more frequent use of latrines at school and home (Table 3). For example, only three (5%) and 19 (31%) out of 62 grade 1 schoolchildren reported to have urinated in the school latrine and defecated in the home latrine the previous day, respectively. In comparison, a higher proportion of latrine use was noted among grade 7 children, with 42 (29%) and 73 (51%) out of 143 children reporting to have used the school latrine for urination and home latrine for defecation the previous day, respectively.

### "Popular knowledge" among schoolchildren of latrines

Among the 24 children participating in the in-depth interviews, only six who lived in the lowland area highlighted latrines as a mean to prevent the spread of diseases or as a way of protecting the environment from "pollution". These six children emphasized that open defecation may cause germs to spread and could make people sick. A grade 7 female child using a latrine at home described the importance of latrine use as to "*avoid germs*". She further explained that "*germs from feces could be transmitted by flies to food and then people could be sick from eating the food*".

Additionally, five children explained that latrines were important to avoid bad smell and dirtiness around the homes and in the community. Among grade 1 children mainly living in the highland (coming mainly from the Xa Phó ethnic minority group), 11 of the 24 children participating in the in-depth interviews could not think of any reason as to why a latrine would be of importance.

### Child perception of hygienic latrines

From the in-depth interviews it was noted that the children perceived a hygienic latrine associated with cleanliness. In their perception, a hygienic latrine should have water to flush and should be cleaned on a regular basis. As explained by a grade 4 male school child *"The floor of the latrine must be clean, urinating and defecating places must be clean, with water to flush, no flies and no fecal matter on the floor"*.

The drawings by the children presenting the school and latrines of their "dreams" clearly indicate that children want to see functional and well-organized latrines with basic facilities. All pictures also indicated wishes for more privacy, with doors on all latrines and wall separations for urinals. In all pictures, good accessibility was also stressed with plenty of space for urination and with more than one chamber available for defecation. Finally, many children depicted hygiene amenities such as water tanks, soap and towels to be of importance to them for a good school latrine (see Additional file 1).

### Child perception about unappealing latrines

According to the children's explanations during the in-depth interviews, an important reason for not using latrines was related to the dirtiness and inhaling "smelly air", both at home and at schools. A range of words were used by the children to describe the status of the school latrines, in particular the smell of urine and feces, such as *rất khai *(a strong smell), *hôi *(smelly) for urine and *thối *(bad smell), *bẩn *(dirty) and *rất bẩn *(very dirty) for feces. A grade 1 male child explained *"My school latrine is smelly, dirty from soil on the floor, much garbage and urine outside the latrine. I have defecated only one time there, very bad smell"*

Lack of access was another important argument used by the older children to explain why school latrines were not preferred for urination. A grade 7 female child explained why she disliked the school latrine "*There are many people waiting to use the latrine and I do not really like to use the school latrine... therefore I and many of my friends come to urinate on the hill (near the school)..."*

### Management of school latrines

Observations at school found that there was a scarcity of water and lack of buckets to collect and store water at latrines with five out of the six schools not having enough water to serve the latrine. It was clear that the need of water for latrines and personal hygiene purposes were inadequately addressed at the time when school latrines were designed and constructed. Through interviews with school staff, it remained unclear who was responsible for the water supply to latrines. "*I do not allow them (the children) to use the latrine because we do not have water... The latrines have had no water from the time the contractor handed it over to us... when I open the door to the latrine, it is extremely dirty, and we cannot make them clean" *(The principal of one secondary school).

The study found that the school management had not been involved in designing the school latrines and that this was the responsibility of external contractors. Some principals mentioned that the contractor who built the latrines was often different from the contractor building the school.

*"The financial source for construction of our school latrine came from the District Construction Management Board. They only requested us to prepare land for construction. The board signed the contract with the contractors. We were allowed to supervise the construction but the Board made the final decision... We did not have the right to participate in the design..." *(The principal of one primary school)

Interviews with the school managers and teachers clearly showed that all six school latrines lacked financial resources to maintain the latrines. There was no separate budget allocated for the maintenance of school latrines. Such costs had to be covered by the annual school budget. No instructions were given on how to maintain and repair the latrines provided to the six schools. The principal of one secondary school explained *"We have no funds to provide toilet paper so the children use sticks for anal cleaning and it results in the latrine getting blocked*".

### Support provided to schoolchildren for latrine use

At the community level, no activity was found, including encouragement by the parents, guidance of children and social practices, to enable the schoolchildren to use latrines, even if the children lived in households with latrines. Open defecation in the field or hiding places such as the bush, animal-pens was seen as acceptable by family members, in particular for small children.

At schools, most teachers found it difficult to teach children how to use a latrine and language barriers made it even more difficult. According to the teachers, it was impossible to teach EMG children (e.g. Xa Phó and Dao groups) how to use a latrine correctly. Furthermore, most school staff agreed that EMG children have a low awareness of latrine use. Thus, they did not believe an EMG child to be able to use a latrine properly. A female teacher in a primary school replied when asked why they did not teach EMG children to clean latrines "*Because they will not be able to... We did not teach them because they are so small/young (bé quá). They are ethnic minority people (người dân tộc). They do not know how to do. They know nothing!"*

Interviews with school staff and parents clearly indicated that there was no communication between the school and home aimed at changing sanitation behavior of the children. In the established parent-child association the main task of adult household members was to focus on helping school staff mobilize the children to attend school, while sanitation practices were never addressed. According to schoolteachers, the family plays the most important role in shaping the sanitation behavior of schoolchildren. A teacher participating in a FGD explained "*Teaching children hygiene behavior should be done by the family.... The school plays an important role but.... Many other factors will make decisions for children and the family context is the most important factor to shape the behavior of the children.... Teaching hygiene behavior for children is the parents' responsibility." *By contrast, the parents believe that guidance on latrine use is the responsibility of the school and its staff as argued by a parent of a secondary school child participating in the FGDs *"The parent (laugh): we have never tried to see the latrine of this school... Our children's activities happening there (at the school) we do not know if they use school latrine... We think our kids learnt to use the latrine at school because it is managed by the school"*

## Discussion

The schools included in this study followed the national programs and standards for sanitation promotion in terms of infrastructure and hygiene education. However, the children, across both genders, ethnic groups and at all ages still found defecation in the open a more compelling alternative and therefore continued with open defecation and urination. The issue of age, ethnicity and the three barriers for improved latrines use of cleanliness status of latrines, the management of school latrines and the teaching approach to sanitation promotion will now be further discussed.

### The difference of latrine use by age group

The present study found that older children reported using latrine more than younger children. It might be explained that young children are often more afraid to use a latrine than older children. They may be unable to open the door, afraid of the dark or the pit. Similarly to a qualitative and quantitative study of 360 households in 9 villages and actors (implementer and household levels) in Bangladesh in 2006 showed the reason for not using latrines of children is that children's faces were considered as less impure in the society. Thus, children feared to go to latrine for defecation rather than they felt comfortable to defecate in open places [15]

### Ethnicity: is it important for school sanitation behavior?

The current study found no clear relationship between the ethnic group affiliation and the custom of open defecation and open urination among children, although one group, the Day, more often reported open defecation and open urination (Table 3). In fact, the Day students in this study had fewer home latrines than the students of other ethnicities, because of the community background. They therefore had no choice but to practice in the open. Also, we found that there was no stigma associated with child open defecation in the study area, which was considered normal and socially accepted practice. This finding is similar to results from studies in rural Tamilnadu [37] and Bangladesh [15], leading to challenges to encourage toilet usage. In future sanitation promotion in the study area, it is therefore important to address the social norms of open defecation, especially parent, who are setting norms for children's sanitation behavior.

### Poor cleaning standards, smell and overcrowding

The reluctance among school children to use the school latrines because of the bad smell noted in this study is consistent with a number of school based studies in other countries. Vernon et al. [7] reported that schoolchildren in Sweden and the United Kingdom avoided using latrines for defecation because the latrines were too dirty. Similarly, toilet habits among 385 Swedish schoolchildren 6-16 years found them to be influenced by their perceptions of offensive smell and the physical appearance of latrines [8]. A few in-depth studies are available on the perception of smell and hygiene in Vietnam. Results emphasize the influence of miasmatic beliefs that smells are permeable to the body and cause diseases [38,39]. One qualitative community study conducted in the same study area found a reluctance across all ethnic minorities to use enclosed latrines as promoted by authorities, as it was perceived that "dirty air" could penetrate the body and make people sick [40]. In our study, "smelly air" from the latrine made schoolchildren reluctant to use the latrine. Similar findings were reported in a qualitative study in Senegal where good smell was a great motivator for toilet compliance behavior among school children [41]. These results underscore the importance of offering appealing school toilets if children are expected to use them consistently.

In this study, five of the six schools followed national standards for latrine infrastructure, i.e. one latrine for a maximum of 200 children [22]. However, during school breaks it was clear that this number of children could not be accommodated in the limited number of latrines; children therefore found alternative sites for defecation and urination. A previous assessment of school sanitation and hygiene education programs in Vietnam found that overcrowding added to the offensive smell at school latrines since children did not have time to clean toilets with water after use [29,42]. The same problem of overcrowding and smelly latrines documented in a school sanitation program in Malawi, led UNICEF to increase the number of separate urinals, which were also cheaper to construct compared with full toilets [43]. Similar recommendations can be made based on the findings in this study, which observed that school urinals are often constructed together with toilets giving rise to a foul smell. Increasing the number of latrines and appropriate design is thus of importance when encouraging children to use latrines at school.

### Appropriate design and maintenance of school sanitation facilities

This study showed that the standard school latrine design used throughout Vietnam are following a blue-print design and not allowing for adaptation to the local context and user preferences of children. In 2002, UNICEF Vietnam promoted a well-lighted, ventilated, easy to maintain and child-friendly school latrine [20,21]. However, so far no study has documented the relevance and uptake of such child-friendly latrines in Vietnam, including in multi-ethnic areas. Based on our observations, a child-friendly school latrine would have to be sufficiently appealing to compete with the current practice of open defecation, where children give preference to fresh air in particular, easy access without waiting time, and privacy.

This study also highlighted that the management of individual schools are not in a position to influence the design, planning and construction of school latrines and, most importantly, do not have budgets allocated for maintenance and cleaning of the sanitation facilities. Interview data from children clearly stressed, that the cleanliness of latrines was not taken care of adequately, despite being a key determining factor for use.

According to WHO guidelines, the process and cost of maintenance of school latrines should be considered during the design and construction phase [2]. Experiences from school based sanitation programs in Bangladesh confirmed that clear roles and responsibility of school management had to be defined early in the construction process to achieve successful sanitation facility management [44]. In Vietnam, the Department of Education is responsible for benchmarking schools on an annual basis, including assessing the sanitary conditions of latrine facilities. However, maintenance mechanisms and indicators of child hygiene behavior (e.g. use of latrines) are currently not included in national surveys.

It is therefore recommended that maintenance of school sanitation facilities and sanitary practices of school children should form an integrated component of annual school assessments. But most importantly, educational authorities must allocate time, efforts and budgets for maintaining and keeping school latrines in a clean and appealing state, if any change of child sanitation behavior is to be expected.

### A new teaching approach to school sanitation promotion

Our study showed that teachers did what they were expected to do at schools following the national curriculum and the national sanitation promotion program. However, the learning process applied at the schools under study was clearly only information-based and did not include practical instruction or guidance of individual schoolchildren and is therefore, likely to be insufficient to facilitate a change in latrine use. It was also found that teachers perceived sanitation promotion a difficult task with EMG children seen as incapable of learning good sanitation behavior.

Educational studies have pointed to the need of both cognitive and social learning of children [45] and cultural competences of teachers in multi-ethnic areas [46]. One study of girl education among four ethnic Vietnamese minorities, confirmed that teachers were indeed more likely to teach children successfully if they understood ethnic minority culture [47]. However, similar to this study, the Vietnamese educational approach is still overwhelmingly lecture-based and non-participatory [47]. The analysis also stated that the quality of education in mountainous and resource poor areas of Vietnam is decreased by the fact that many teachers cannot teach in the mother tongue of the EMGs, that curriculum often does not draw on local examples and that teacher's qualifications are poor [48].

In accordance, this study points toward a clear need to educate teachers to perform sanitation promotion that is contextual, specific and practical rather than theoretical. Likewise, the teaching should to a greater degree involve the schoolchildren, be adapted to the different age groups and support bilingual education. As supported by this study, teachers need to acknowledge that the ethnicity of children may not be a determining factor for use of sanitation and good sanitation behavior. Rather, poverty, access to toilets at home and the poor conditions and overcrowding of school toilets are of great importance when encouraging children to use a latrine.

The present study also found that school-based sanitation promotion efforts were implemented separately from community based activities and not involving parents. Improved links between school and home can is expected to support that schoolchildren can apply at home what they have learnt at school, thus contributing to the effectiveness of school-based sanitation promotion [2]. The study communes already had student-parent associations which could form an effective communication link and channel for motivation from school to community and home as suggested by the WHO [2]. Other relevant bodies that could facilitate contact between school and home are teacher-parent associations, where teachers can discuss with the parents the importance of guiding children to practice good sanitation behavior at home, e.g. using a latrine and washing hands after defecation.

### Limitations of the study

There were limitations to the present study. As it was done in only two communes, the results may not be representative for other ethnic groups and mountainous areas in Vietnam - especially poorer ethnic minority communities placed in high mountains far away from urbanized settings can be expected to have even poorer access to sanitation at home and at schools.

The study did not go into details with identifying appropriate school and home based child sanitation promotion methods. It is therefore suggested that further studies focus on how to promote good latrine maintenance at schools, which teaching methods can be used to encourage children to use latrines, and how to link home and school based sanitation promotion.

## Conclusions

The paper shows that the current school based sanitation promotion is insufficient to change sanitation behavior of school children irrespective of their ethnic origin. It is important that schools, households and communities work more closely together to increase uptake of latrine use among schoolchildren. Also, the contractors of latrines facilities must work more closely with local school management when constructing latrines, including identifying location, design and appropriate systems of water supply. A separate budget needs to be allocated to allow the school to maintain the sanitation infrastructure and keep it hygienic and appealing for users. Also, teachers need to acknowledge the importance of offering clean and appealing school sanitation facilities for supporting good child sanitation behavior.

## Competing interests

The authors declare that they have no competing interests.

## Authors' contributions

LTTX was responsible for the study design and implementation, data analysis, and drafting the manuscript. FK reviewed the study design and manuscript revisions. AD, LNH and TTR participated in the data analysis and manuscript revisions. All authors read and approved the final manuscript.

## Pre-publication history

The pre-publication history for this paper can be accessed here:

http://www.biomedcentral.com/1471-2458/12/140/prepub

## Supplementary Material

Additional file 1 **A dream of school latrine made by a schoolchild with more space, more water, separate for girls and boys, slogan on remind using latrine, no smoking**. Click here for file
