# Supplementary figures and images for: Sanitation behavior among schoolchildren in a multi-ethnic area of Northern rural Vietnam
Source: BMC Public Health. 2012 Feb 21;12:140. doi: 10.1186/1471-2458-12-140 (PMC3305623; doi:10.1186/1471-2458-12-140)

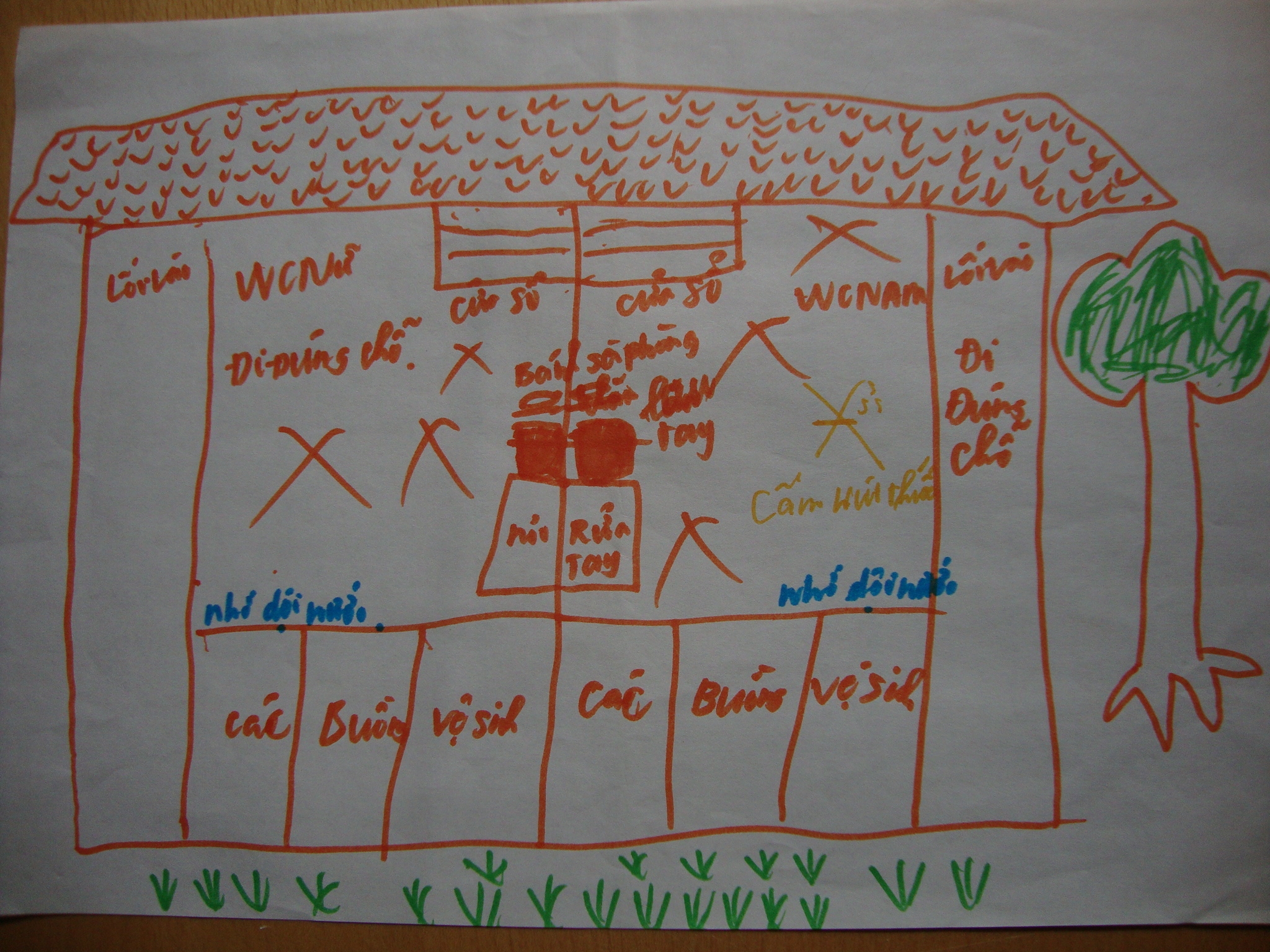

Supplement: Additional file 1 — A dream of school latrine made by a schoolchild with more space, more water, separate for girls and boys, slogan on remind using latrine, no smoking. [file 1471-2458-12-140-S1.JPEG]
